# The complete chloroplast genome sequence of *Laplacea alpestris* and its phylogenetic position

**DOI:** 10.1080/23802359.2020.1837687

**Published:** 2020-11-22

**Authors:** Xiang-Qin Yu, Qiong Zhang, Yin-Zi Jiang, Hua Peng, Jian-Li Zhao, Shi-Xiong Yang

**Affiliations:** a CAS Key Laboratory for Plant Diversity and Biogeography of East Asia, Kunming Institute of Botany, Chinese Academy of Sciences, Kunming, P. R. China; b Laboratory of Ecology and Evolutionary Biology, State Key Laboratory for Conservation and Utilization of Bio-Resources in Yunnan, Yunnan University, Kunming, P. R. China; c Yunnan Key Laboratory of Plant Reproductive Adaption and Evolutionary Ecology, Yunnan University, Kunming, P. R. China

**Keywords:** *Laplacea alpestris*, chloroplast genome, Illumina sequencing, phylogenetic analysis

## Abstract

*Laplacea alpestris* is a member of the genus *Laplacea*, which distributes in Central and South America. Genetic information of *L. alpestris* would provide guidance for the phylogenetic position of this species. Here, we reported and characterized its complete chloroplast (cp) genome using Illumina pair-end sequencing data. The total chloroplast genome size of this species was 157,211 bp, including inverted repeats (IRs) of 26,103 bp, separated by a large single copy (LSC) and a small single copy (SSC) of 86,749 and 18,256 bp, respectively. A total of 132 genes, including 37 tRNA, 8 rRNA, and 87 protein-coding genes were identified. Phylogenetic analysis showed that *L. alpestris* formed a monophyletic clade with *Laplacea fruticosa*, and then grouped with *Apterosperma oblata*. The systematic position of Southeast Asian *Laplacea* species needs further studies.

*Laplacea* Kunth, with ca. 30 species, mainly distributed in South and Central America, Malaya, Indonesia (Kobuski 1949, 1950). The genus was built in 1822 based on the type species (*L. speciosa* Dyer) from Peru (Humboldt et al. 1822). The systematic position of *Laplacea* changed significantly among different taxonomic treatments based on morphological and floral ontogenic evidence, and even was included in *Gordonia* s.l. (Airy-Shaw 1936; Sealy 1958; Keng 1962; Ye 1990; Tsou 1998). Molecular phylogenetic analysis based on *rbcL* and *matK*, and the chloroplast genome sequences suggested that only *Gordonia brandegeei* H. Keng nom. nov. (=*Laplacea grandis*) (Keng 1980) should be retained in *Gordonia* s.s., other species from *Laplacea* were members of Theeae (Prince and Parks 2001; Yu et al. 2017). However, only scarce species were included in previous studies and only one chloroplast genome was reported for *Laplacea* (*Laplacea fruticosa*, Yu et al. 2017). In this study, we present the complete chloroplast genome sequence of *Laplacea alpestris* Dyer using Illumina sequencing technology.

Leaf sample of *L. alpestris* was obtained from the Herbarium of University of Florida (FLAS, voucher FLAS 180,103), the specimen was collected from Massif de la Selle of Haiti. Genomic DNA was isolated using a modified CTAB approach (Doyle and Doyle 1987). The 150 bp pair-end reads were sequenced based on the Illumina Hi-Seq 2500 platform. Totally, 14,086,309 reads in size of 4.71 G were obtained for the next analysis. The chloroplast genome was *de novo* assembled by GetOrganelle script (Jin et al. 2020), with SPAdes version 3.10.1 as assembler (Bankevich et al. 2012), and visualized the paths of the cp genome using Bandage version 0.8.1 (Wick et al. 2015). Geneious version 8.0.2 (Kearse et al. 2012) was used to annotate the *L. alpestris* and then submit to Genebank (the accession number is MT916289). The size of chloroplast genome of *L. alpestris* is 1,57,211 bp. The GC content of the genome is 37.2%. The length of inverted repeats (IR), large single copy (LSC), and small single copy (SSC) were 26,103, 86,749, and 18,256 bp, respectively. The chloroplast genome of *L. alpestris* contained 132 genes, with 8 rRNA genes, 37 tRNA genes, and 87 protein-coding genes. Annotation revealed that 4 rRNA genes, 7 tRNA genes, and 7 protein-coding genes were duplicated in the IR region.

To confirm the phylogenetic position of *L. alpestris*, we conducted the phylogenetic analysis by combining the chloroplast genome of *L. alpestris* and other 55 species (including 50 ingroups from Theaceae and 5 outgroups). Sequences were aligned using MAFFT version 7.407 (Katoh and Standley 2013) with the Auto algorithm. RAxML (Stamatakis 2014) was used to build a maximum likelihood (ML) tree, and bootstrap support (BS) were calculated using 1000 replicates. The maximum likelihood phylogenetic tree revealed that *L. alpestris* and *L. fruticosa* formed a monophyletic clade (BS = 100%), which was closely related to *Apterosperma oblata* (Figure 1). However, only species of *Laplacea* from Central and South America were studied till now (i.e. *L. alpestris*, *Laplacea fruticosa*, and *Laplacea portoricensis*) (Prince and Parks 2001; Yu et al. 2017), whether species from Southeast Asia will fall into *Laplacea* or *Gordonia* s.s. need further researches. The complete chloroplast genome of *L. alpestris* would be useful for the genetic diversity studies of this species and provided new molecular data to illuminate the phylogenetic relationships within Theaceae.

**Figure 1.**
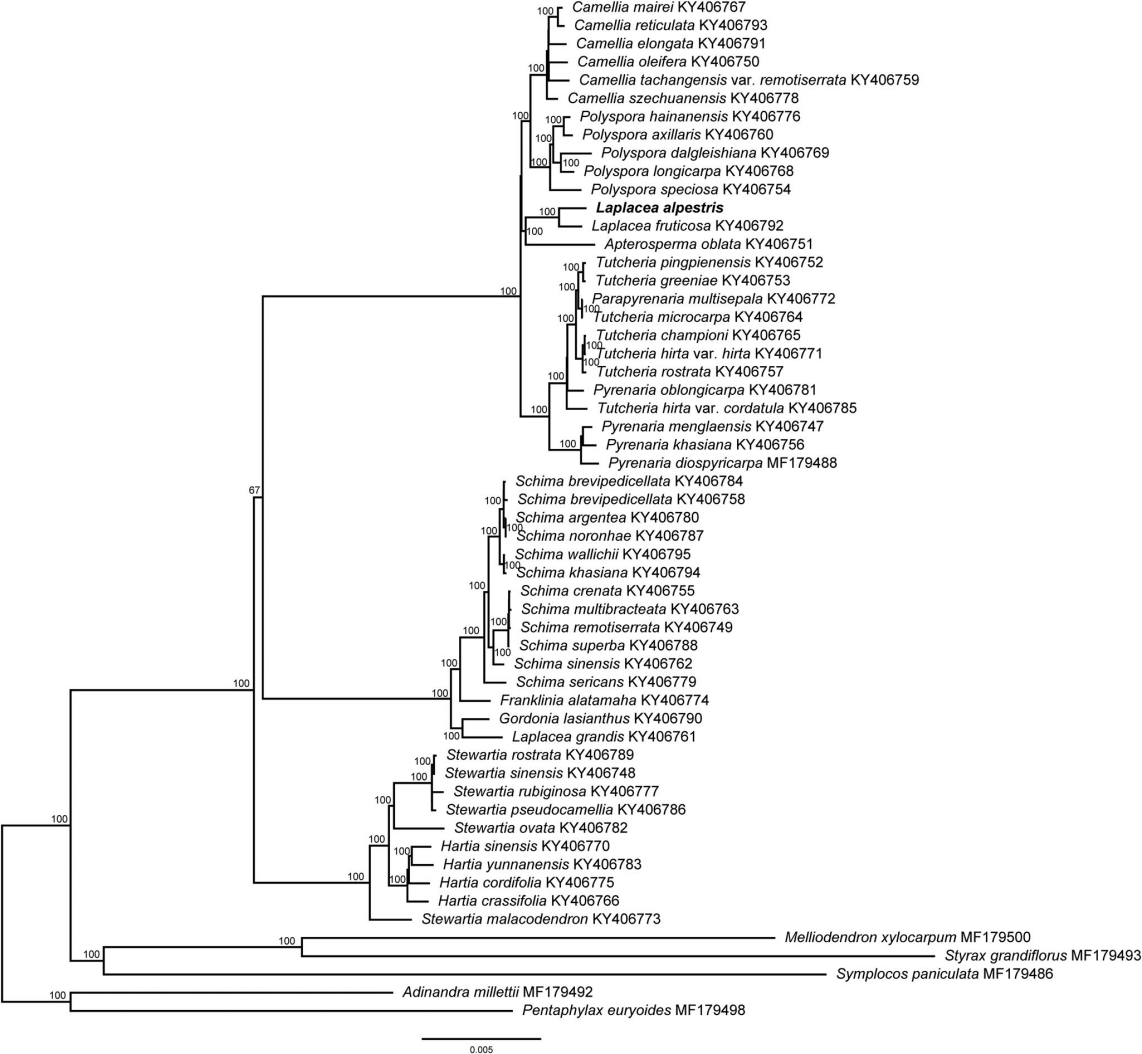
Maximum likelihood tree of Theaceae based on 55 complete chloroplast genome sequences, including *Laplacea alpestris* (GenBank ID: MT916289) sequenced in this study. The bootstrap support values are shown beside the nodes. Five representative taxa of Styracaceae (*Melliodendron xylocarpum*, MF179500; *Styrax grandiflorus* MF179493), Symplocaceae (*Symplocos paniculata*, MF179486), and Pentaphylacaceae (*Adinandra millettii* MF179492; *Pentaphylax euryoides* MF179498) from Ericales were used as outgroups.

## Data Availability

The data that support the findings of this study are openly available in GenBank of NCBI at https://www.ncbi.nlm.nih.gov, reference number MT916289.
